# Genome Sequence Analysis Reveals Evidence of Quorum-Sensing Genes Present in Aeromonas hydrophila Strain M062, Isolated from Freshwater

**DOI:** 10.1128/genomeA.00100-15

**Published:** 2015-03-12

**Authors:** Kok-Gan Chan, Wen-Si Tan, Chien-Yi Chang, Wai-Fong Yin, Nina Yusrina Mumahad Yunos

**Affiliations:** a Division of Genetics and Molecular Biology, Institute of Biological Sciences, Faculty of Science, University of Malaya, Kuala Lumpur, Malaysia; b Interdisciplinary Computing and Complex BioSystems (ICOS) Research Group, School of Computing Science, Newcastle University, Newcastle upon Tyne, United Kingdom; c The Centre for Bacterial Cell Biology, Medical School, Newcastle University, Newcastle upon Tyne, United Kingdom

## Abstract

Aeromonas hydrophila has emerged worldwide as a human pathogen. Here, we report the draft whole-genome sequence of a freshwater isolate from Malaysia, A. hydrophila strain M062, and its *N*-acylhomoserine lactone genes are also reported here.

## GENOME ANNOUNCEMENT

Quorum sensing (QS) is a term coined by Fuqua and coworkers to define the phenomenon of a bacterial cell-to-cell communication system that controls bacterial physiological processes in a population density-dependent manner (1). This process describes the event whereby an increase in the population density of the bacterial cell is proportional to an increase in the concentration of signal molecule(s) in the extracellular environment (2, 3). Aeromonas hydrophila is QS bacterium and is a known pathogen, as it can cause minor skin infections and gastroenteritis in humans (4, 5). In this study, we report the whole-genome sequence and the QS genes of A. hydrophila strain M062.

A. hydrophila M062 was isolated from the Sungai Tua waterfall, Selangor, Malaysia. The genomic DNA of A. hydrophila M062 was extracted using the MasterPure DNA puriﬁcation kit (Epicentre, Inc., Madison, WI, USA). A Qubit 2.0 fluorometer (Invitrogen, USA) and NanoDrop spectrophotometer (Thermo Scientific, USA) were used to quantify and qualify the DNA preceding whole-genome shotgun sequencing on an Illumina MiSeq personal sequencer (Illumina, Inc., San Diego, CA, USA) and generated 4,984,734 paired-end reads. Subsequently, the paired-end reads were trimmed and *de novo* assembled with CLC Genomics Workbench version 5.1 (CLC bio, Denmark). Gene prediction was conducted using Prodigal (version 2.60) (6), followed by gene annotation using RAST (7). Next, tRNAs and rRNAs were predicted with tRNAscan-SE version 1.21 (8) and RNAmmer (9), respectively.

From this whole-genome sequencing, a total of 4,974,350 reads were generated. After trimming (1,548,021 quality reads), the reads were assembled into 140 contigs, with an *N*_50_ of 93,561 and an average coverage of 51.4-fold. The G+C content of the draft genome of A. hydrophila strain M062 is 60.5%. An analysis of its genome sequences led to the identification of 4,392 coding DNA sequences (CDSs), 95 tRNAs, two copies of 5S rRNA, and one copy each of 16S rRNA and 23S rRNA.

The annotation result showed that the *luxI* and *luxR* homologues of A. hydrophila strain M062 are located at contig 35. With the availability of the whole-genome sequence of A. hydrophila strain M062, it may provide a better understanding of the QS system in this strain.

### Nucleotide sequence accession numbers.

This whole-genome shotgun project has been deposited at DDBJ/EMBL/GenBank under the accession no. JSXE00000000. The version described in this paper is the first version, JSXE01000000.
